# COVID-Classifier: an automated machine learning model to assist in the diagnosis of COVID-19 infection in chest X-ray images

**DOI:** 10.1038/s41598-021-88807-2

**Published:** 2021-05-10

**Authors:** Abolfazl Zargari Khuzani, Morteza Heidari, S. Ali Shariati

**Affiliations:** 1 Department of Electrical and Computer Engineering, University of California, Santa Cruz, Santa Cruz, CA USA; 2 School of Electrical and Computer Engineering, The University of Oklahoma, Norman, OK USA; 3 Department of Biomolecular Engineering, University of California, Santa Cruz, Santa Cruz, CA USA

**Keywords:** Medical research, Mathematics and computing

## Abstract

Chest-X ray (CXR) radiography can be used as a first-line triage process for non-COVID-19 patients with pneumonia. However, the similarity between features of CXR images of COVID-19 and pneumonia caused by other infections makes the differential diagnosis by radiologists challenging. We hypothesized that machine learning-based classifiers can reliably distinguish the CXR images of COVID-19 patients from other forms of pneumonia. We used a dimensionality reduction method to generate a set of optimal features of CXR images to build an efficient machine learning classifier that can distinguish COVID-19 cases from non-COVID-19 cases with high accuracy and sensitivity. By using global features of the whole CXR images, we successfully implemented our classifier using a relatively small dataset of CXR images. We propose that our COVID-Classifier can be used in conjunction with other tests for optimal allocation of hospital resources by rapid triage of non-COVID-19 cases.

## Introduction

Computed tomography (CT), lung ultrasound (LUS), and Chest-X ray (CXR) radiography are among the most commonly used imaging modalities to identify COVID-19 infections^1–3^. Compared to other modalities, chest X-ray radiography is a low-cost, easy-to-operate, and low radiation dose clinical screening method^1–3^. CXR radiography is one of the most commonly used and accessible methods for rapidly examining lung conditions^4^. CXR images are almost immediately available for analysis by radiologists. CXR radiography's availability made it one of the first imaging modalities to be used during the recent COVID-19 pandemic. In addition, the rapid CXR turnaround was used by the radiology departments in Italy and the U.K. to triage non-COVID-19 patients with pneumonia to allocate hospital resources efficiently^2^. However, there are many common features between medical pneumonia and COVID-19 images caused by other viral infections such as common flu (Influenzas A)^5^. This similarity makes a differential diagnosis of COVID-19 cases by expert radiologists challenging^5,6^. A reliable automated algorithm for the classification of COVID-19 and non-COVID-19 CXR images can speed up the triage process of non-COVID-19 cases and maximize the allocation of hospital resources to COVID-19 cases.

Machine learning (ML) based methods have shown unprecedented success in the reliable analysis of medical images^7–11^. ML-based approaches are scalable, automatable, and easy to implement in clinical settings^12,13^. A common application of ML-based image analysis is the classification of images with highly similar features. This approach relies on the segmentation of image region of interest, identification of effective image features extracted from the segmented area in the spatial or frequency domain, and development of an optimal machine learning-based classification method to accurately assign image samples into target classes^14^. Recently, several ML-based methods for the diagnosis of COVID-19 medical images has been proposed^1–3,15^. Wang et al.^3^ applied a pre-trained deep learning model called DenseNet 121 to CT images aiming to classify COVID-19 imaging tests into positive and negative categories leading to 81.24% accuracy. Also, Roy et al.^2^ studied the application of deep learning models to analyze COVID-19 infections in a small dataset of lung ultrasonography(LUS) images (only 11 patients). Zhang et al.^15^ proposed the application of the lung-lesion segmentation in CT images a ResNet-18 classifier model for three classes of COVID-19, pneumonia, and normal, generating an accuracy of 92.49%.

Here, we hypothesized that CXR images of COVID-19 patients can be reliably distinguished from other forms of pneumonia using an ML-based classifier. We used a dimensionality reduction approach to generate a model with an optimized set of synthetic features that can distinguish COVID-19 images with an accuracy of 94% from non-COVID-19 cases. A distinct feature of our model is the identification and extraction of features from the whole CXR image without any segmentation process on chest lesions. This new quantitative marker not only enables us to avoid segmentation errors but also reduces the computational cost of our final model. Our study provides strong proof of concept that simple ML-based classification can be efficiently implemented as an adjunct to other tests to facilitate differential diagnosis of CXR images of COVID-19 patients. More broadly, we think that our approach can be easily implemented in any future viral outbreak for the rapid classification of CXR images.

## Results

### Generation of synthetic features

**Figure 1:**
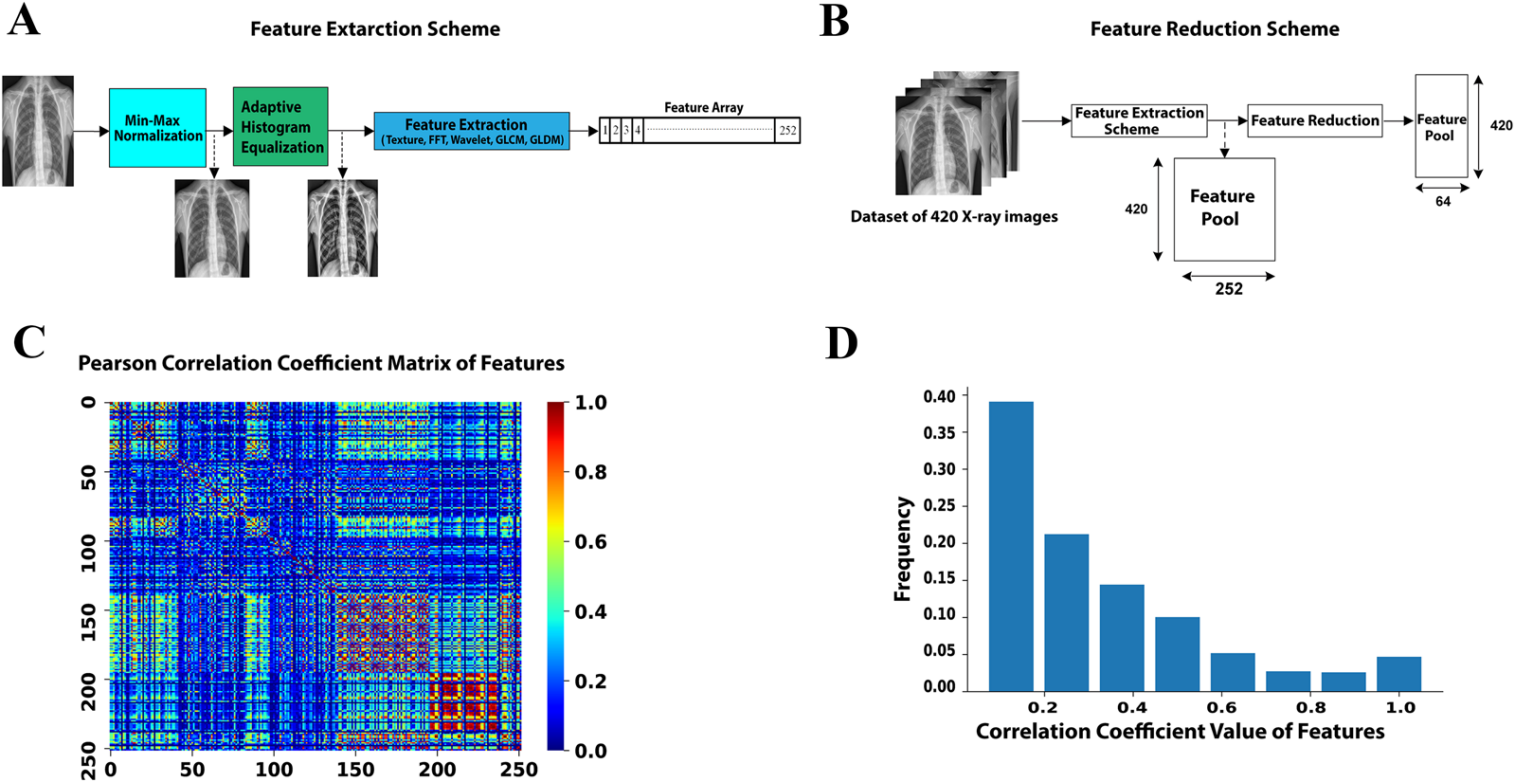
**(A)** Feature extraction schematic diagram to build a feature array for each CXR image using the Texture, FFT, Wavelet, GLCM, and GLDM methods (See method section for the description of the features). **(B)** A schematic diagram of creating a feature pool for 420 CXR images and applying a feature reduction method. **(C,D)** Correlation analysis of features. The heat map **(C)** and histogram representation **(D)** of the Pearson correlation coefficients.

Identification of optimal features of the CXR images can decrease the feature space of ML models by generating key correlated synthetic features and removing less important features. These synthetic features perform more reliably in classification tasks while reducing the size of the ML models. Importantly, a more robust ML classifier can be generated by decreasing the ratio between the number of image features and the number of training data cases per class. We initially extracted 252 features from the whole CXR image without involving lesion segmentation (Fig. 1A and Supplementary Figure 1) to finally generate a feature pool from 420 CXR images (Fig. 1B). We hypothesized that we can use a feature analysis scheme to find an optimal number of features and reduce the size of the feature space. Figure 1C shows the pairwise feature association by Pearson correlation coefficients matrix obtained from 252 features. An analysis of the initial feature pool's histograms reveals that more than 73% of features have correlation coefficients of less than 0.4 (Fig. 1D), confirming a comprehensive view of the cases with relatively small redundancy. We used Kernel-Principal Component Analysis (PCA) method to decrease the size of the feature space to an optimal number of synthetic features composed of correlated features. By employing PCA, we converted the original pool of 252 features to 64 new synthetic features resulting in a ~ 4 × smaller feature space. We used this 64-element feature vector in the final classification process.

### Classification performance

**Figure 2:**
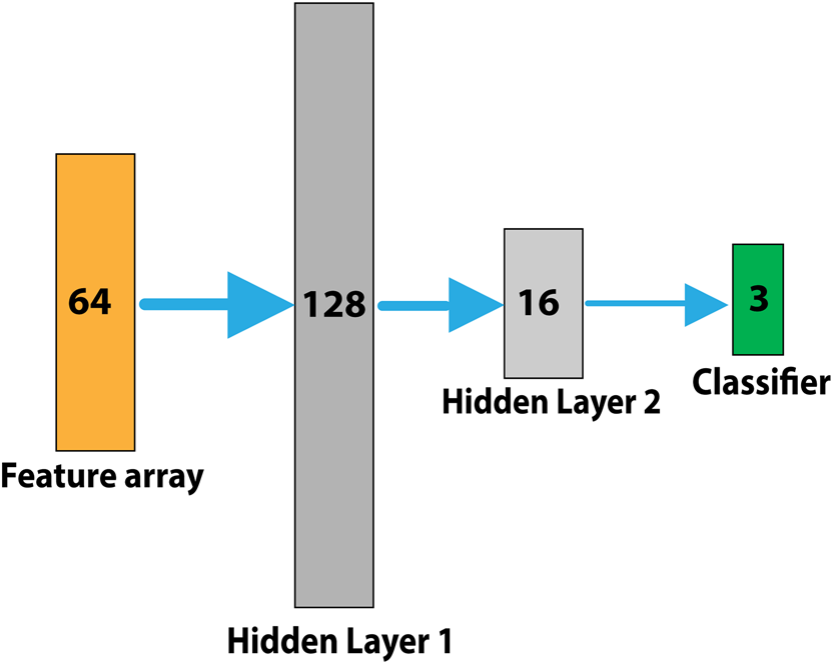
Neural network classifier including two hidden layers of 128 and 16 neurons respectively, followed by a final classifier to classify cases into three categories of normal, COVID-19, non-COVID-19 pneumonia.

To design our classifier, we grouped our CXR images into three target classes, each containing 140 images; normal, COVID-19, non-COVID-19 pneumonia (Supplementary Figure 2). We trained a multi-layer neural network, including one output classifier layer and two hidden layers, aiming to classify CXR images into three target groups (Fig. 2).

**Figure 3:**
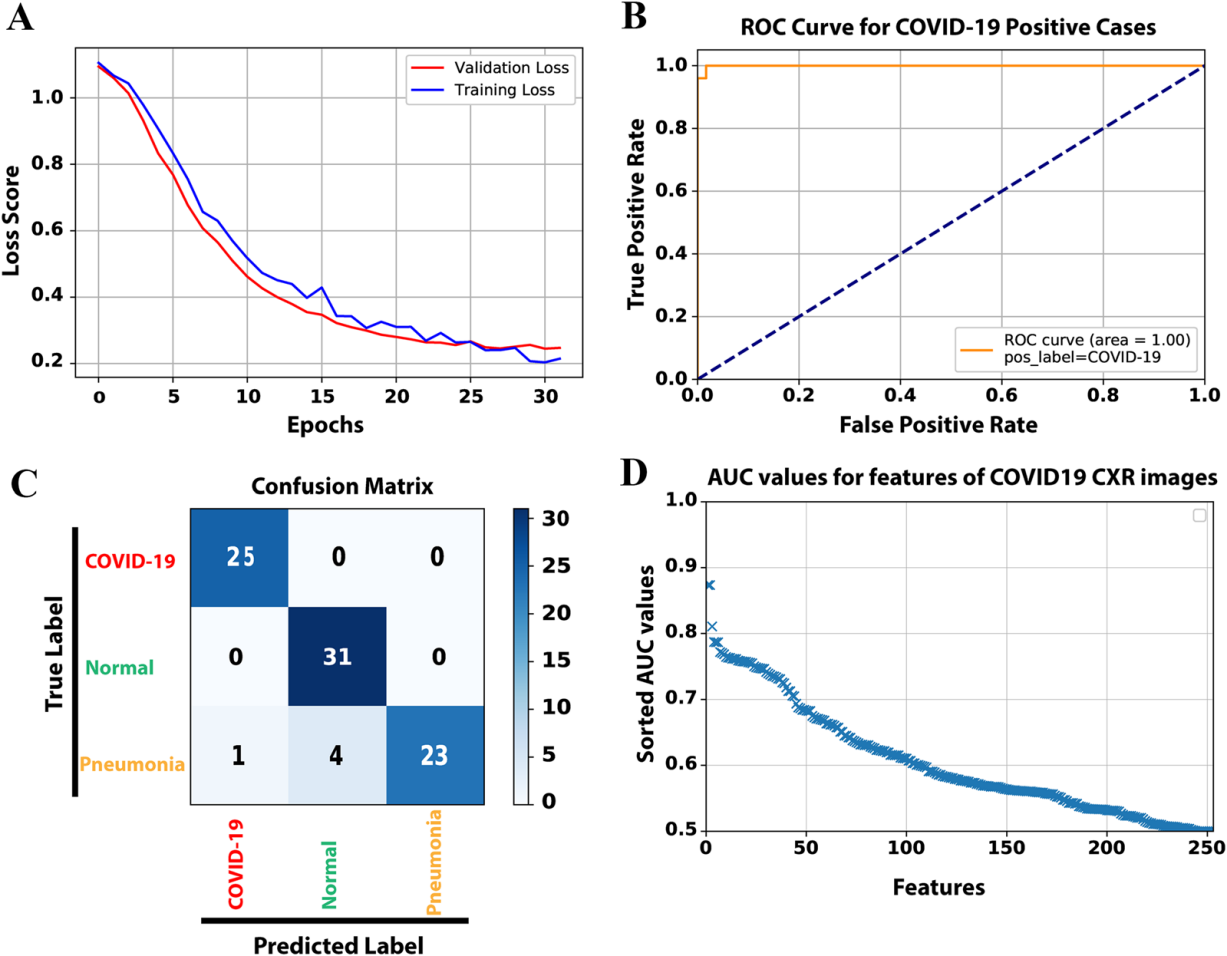
**(A)** The loss score graph of the training and validation sets during the model training process. **(B)** The ROC curve, generated from 84 test samples, while COVID-19 is assigned as the positive class. **(C)** The Confusion matrix of predicting 84 test samples in three categories. **(D)** To compare and analyze the discrimination power of different single features among the original 252 extracted features, we used AUC values as an indicator. All features were sorted in the order of their AUC values.

After 33 epochs of the training process, both training and validation loss scores reached ~ 0.22, corresponding the accuracy of 94%(Fig. 3A). The loss graph showed a good fit between validation and training curves, confirming that our model is not suffering from overfitting or underfitting. We would like to note that our model has ~ 10,000 parameters that are considerably smaller than typical image classification models such as AlexNET with 60 million parameters^16^, VGG-16 with 138 million^17^, GoogleNet-V1 with 5 million^18^, and ResNet-50 with 25 million parameters^19^. Next, we generated a receiver operating characteristic (ROC) curve and computed the area under the ROC (AUC) to further evaluate the performance of our model (Fig. 3B). A comparison of CXR images of COVID-19 cases with non-COVID-19 showed that our model has100% sensitivity and 96% precision when evaluated on a test set of 84 CXR images (Fig. 3C and Table 1). Moreover, our synthetic feature classifier outperforms any single feature classifier as measured by AUC (Fig. 3D). It is noteworthy that single synthetic features as the primary fast and low computational cost classifier can be accurate up to ~ 90% (Supplementary Figure 3).

**Table 1:** Assessment of evaluation metrics for three target class labels using 84 test samples.

	Precision	Sensitivity	F-score	Support
COVID-19	96%	100%	0.98	25
Normal	89%	100%	0.94	31
Pneumonia	100%	82%	0.91	28

## Discussion

In this study, we proposed an efficient machine-learning classifier that accurately distinguished COVID-19 CXR images from normal cases and pneumonia caused by other viruses. Among different imaging modalities^20–22^, X-ray is still the fastest and prevalent screening tool for detecting lung diseases and infections. However, there are some suspicious lung infection masses in x-ray images, which may result in misdiagnosis. Thus, a new approach to assist in automated lung screening analysis and facilitate the classification of different types of lung diseases is crucial. Our work shows that this is possible with relatively straightforward machine learning classifiers. Our proposed machine learning approach has the following distinctive characteristics:

First, by deriving the global image features from the entire chest area, we avoided the lesion segmentation complexities and errors. In addition, we confirmed that the diagnostic information can be distributed on the entire chest area of the X-ray image, not only in the lesion area.

Second, in the feature extraction scheme, we focused on features obtained from both the spatial domain (Texture, GLDM, GLCM) and frequency domain (Wavelet and FFT), unlike many previous machine learning models analyzing only the texture-based features in the spatial domain. In addition, using the two-class classification results shown in Supplementary Figure 3 (second row), we showed that if we, in an experiment, aim at distinguishing COVID-19 cases from other categories, the discrimination power and performance of features obtained from the frequency domain (FFT group) are more effective than features extracted from the spatial domain. The average AUC of the FFT group is around 0.71, showing the significance of acquiring such frequency domain features compared to other groups with an average AUC value of less than 0.63. Furthermore, the examination of every single feature in this experiment revealed that all top seven features belonged to the FFT category with an AUC value higher than or equal to 0.77, which may indicate that those frequency domain features were more relevant to the detection of COVID-19 cases.

Third, we investigated the influences of applying a dimensionality reduction method to obtain optimal and more correlated features. Interestingly, the results demonstrated that our dimensionality reduction method, in addition to reducing the dimension of feature space, is able to identify the new smaller feature fusion with more correlated information and a lower amount of redundancy. Besides, decreasing the ratio of the number of features to the number of cases per class will improve the reliability and robustness of the ML classifier while decreasing the risk of overfitting. Therefore, we could successfully classify CXR images using a relatively small image dataset of 420 cases. Typically, this is not possible with conventional deep learning models as they need a large dataset.

Although we obtained promising results, there are a few limitations in this study. First, our CXR dataset has a relatively small size. A larger dataset consisting of the cases from different institutions would be useful to more verify our proposed model's robustness and reliability. Also, in our future work, we will investigate different feature selection and feature reduction methods such as DNE^23^, Relief^24^, LPP^5^, Fast-ICA^25^, recursive feature elimination^26^, variable ranking techniques^27^, or merging them with our feature reduction approach. Besides, although the neural network-based classifier utilized in this investigation can solve our complicated problem efficiently, it might be useful to explore other efficient and prevalent classifiers such as SVM^28^, GLM^29^, Random Forest^30^.

## Method

### Dataset and code (GitHub page)

Our Python scripts and dataset are available for download on our GitHub page https://github.com/abzargar/COVID-Classifier.git.

This resource is fully open-source, providing users with Python codes used in preparing image datasets, feature extraction, feature evaluation, training the ML model, and evaluation of the trained ML model. We used a dataset, which is collected from two resources of^31,32^. Our collected dataset included 420 2-D X-ray images in the Posteroanterior (P.A.) chest view, classified by valid tests to three predefined categories of Normal (140 images), pneumonia (140 images), and COVID-19 (140 images). We set all image sizes to 512 × 512 pixels. Supplementary Figure 2 shows three example images.

### Feature extraction

In the scheme that we employed in the feature extraction part (Fig. 1A and Supplementary Figure 1), a total of 252 spatial and frequency -domain features were computed and categorized into five groups of (1) Texture^33^, (2) Gray Level Difference Method (GLDM)^11^ (3), Gray-Level Co-Occurrence Matrix (GLCM)^34^, (4) Fast Fourier Transform (FFT)^35^, and (5) Wavelet Transforms (WT)^36^. Wavelet transforms were decomposed in eight sub-bands. GLDM and GLCM coefficients were also computed in four directions. As illustrated in Supplementary Figure 1, each group or each group-subsection then was passed to a feature calculator function to calculate 14 statistical features comprising of Skewness, Kurtosis, Mean, Entropy, Energy, Std, Mean, Median, Max, Min, Mean Deviation, RMS, Range, MeanGradient, StdGradient, and Uniformity. The feature extraction scheme resulted in 252 features for each X-ray image in total (14 features for Texture, 14 features for FFT, 56 features for GLCM, 56 features for GLDM, and 112 features for Wavelet).

### Evaluation of classification power of extracted features

Supplementary Figure 3A shows the AUC values of single features based on their AUC values in sorted order (highest to lowest) and considering three positive class labels. We used the AUC value as an index to compare the classification power of every single feature. As seen in all three AUC graphs, most of the features reported AUC values of higher than 0.6, where features MeanDeviation_GLDM, Max_FFT, and Kurtosis_Wavelet were the best features associated with positive class labels of Normal, COVID-19, and Pneumonia with an AUC value of 0.91, 0.87, and 0.88, respectively.

Supplementary Figure 3B also compares the performance of five groups of features based on their average AUC values showing there is no significant difference between them, particularly where the positive label is pneumonia. Given COVID is the target class, the FFT group recorded the best performance, while the best group for the Normal class is GLDM.

### Model training and test process

A schematic diagram of our model training and test processes is shown in Supplementary Figure 4. We randomly split the original image dataset into a training set (80%) and a test set (20%). The train-test split is a technique used to evaluate supervised machine learning algorithms' performance where we have the inputs and desired output labels. The machine-learning algorithm uses the training set to make the model learn the patterns in the input by minimizing the error between predictions and target outputs. The test set is then used to evaluate the trained model's performance. Without providing a large enough training dataset, the model cannot generalize the knowledge from the training set to the test set, leading to low predictive accuracy in the test phase for unseen cases, as shown in Supplementary Figure 5.

We chose Adam optimizer to optimize model weights and minimize the categorical cross-entropy loss function. The learning algorithm hyperparameters were set as follows: MaxEpochs = 100, BatchSize = 2, LearningRate = 0.001, ValidationRatio = 0.2, TestRatio = 0.2, TrainRatio = 0.6, and DropoutValue = 0.2. We also used the Early Stopping technique to stop training when the validation score stops improving, aiming to avoid learning algorithm from overfitting. The run-time of different parts of our proposed machine learning scheme, listed in Table 2, indicates that our model needed a short time of 15.4 s to learn training set and 2.03 s to predict one test sample.

**Table 2:** Run-time analysis on the local system with the CPU of Intel Core i7-8750H 2.2 GHz and GPU of RTX2080 Max-Q.

	Training phase	One single predict phase		
		Feature extraction (Fig. 1A)	Feature reduction (Fig. 1B)	Classifier (Fig. 2)
Run-time (s)	15.4	1.98	0.02	0.03

## Supplementary Information

Supplementary Figures.
