# Supplementary material for: COVID-Classifier: an automated machine learning model to assist in the diagnosis of COVID-19 infection in chest X-ray images
Source: Sci Rep. 2021 May 10;11:9887. doi: 10.1038/s41598-021-88807-2 (PMC8110795; doi:10.1038/s41598-021-88807-2)
Supplement: Supplementary file 1 — Supplementary Figures. [file 41598_2021_88807_MOESM1_ESM.pdf]

COVID-Classifier: An automated machine learning model to assist in the diagnosis of COVID-19 infection in chest x-ray images

Abolfazl Zargari Khuzani[1], Morteza Heidari[2], S. Ali Shariati[3,*]

1. Department of Electrical and Computer Engineering, University of California, Santa Cruz, Santa Cruz, CA
abzargar@ucsc.edu
2. School of Electrical and Computer Engineering, The University of Oklahoma, Norman, OK
morteza.heidari@ou.edu
3. Department of Biomolecular Engineering, University of California, Santa Cruz, Santa Cruz, CA
alish@ucsc.edu

*Correspondence: alish@ucsc.edu

Supplementary Figures:

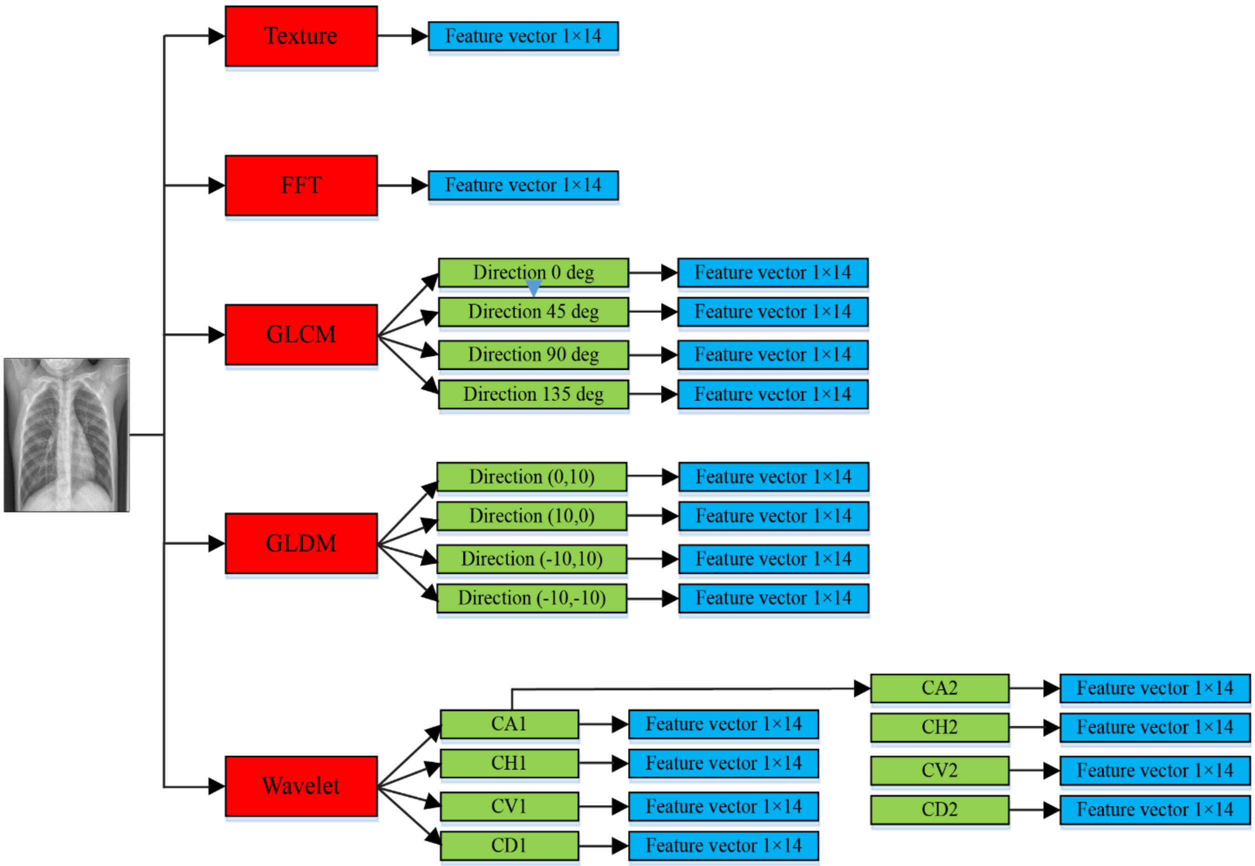

Supplementary Figure 1: The proposed feature extraction schematic diagram

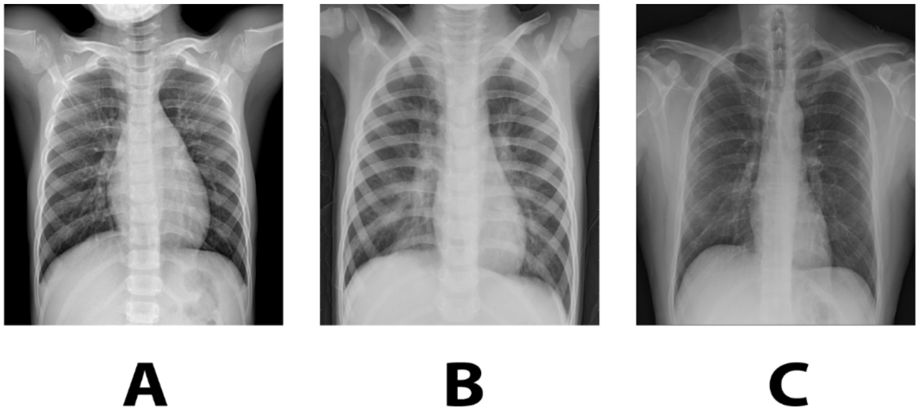

Supplementary Figure 2: Examples of A) Normal, B) Pneumonia, and C) COVID-19

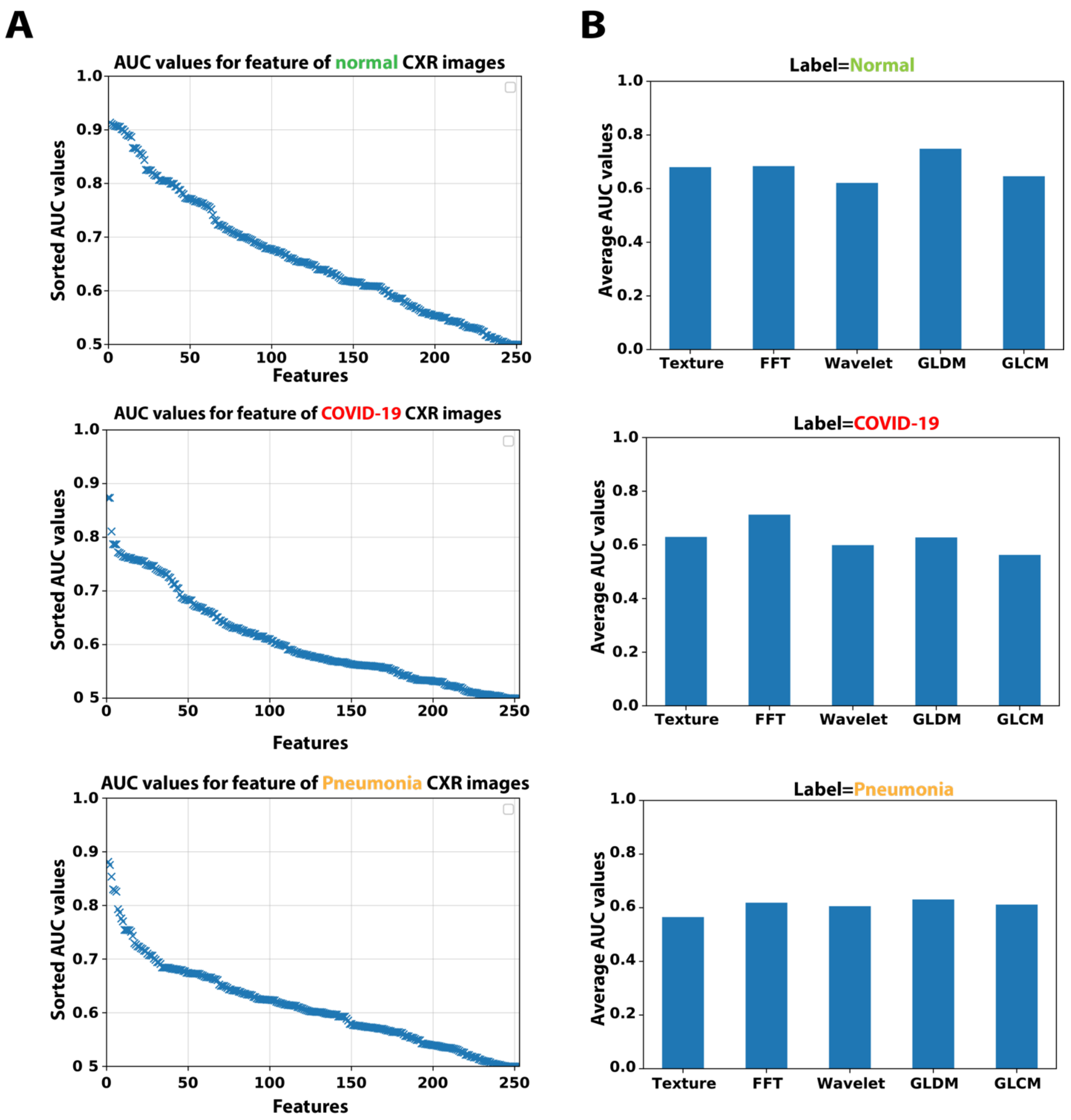

Supplementary Figure 3: A) The performance of every single feature based on the sorted AUC values for three positive class labels. B) The five feature groups for three target classes, compared with each other based on the average AUC values

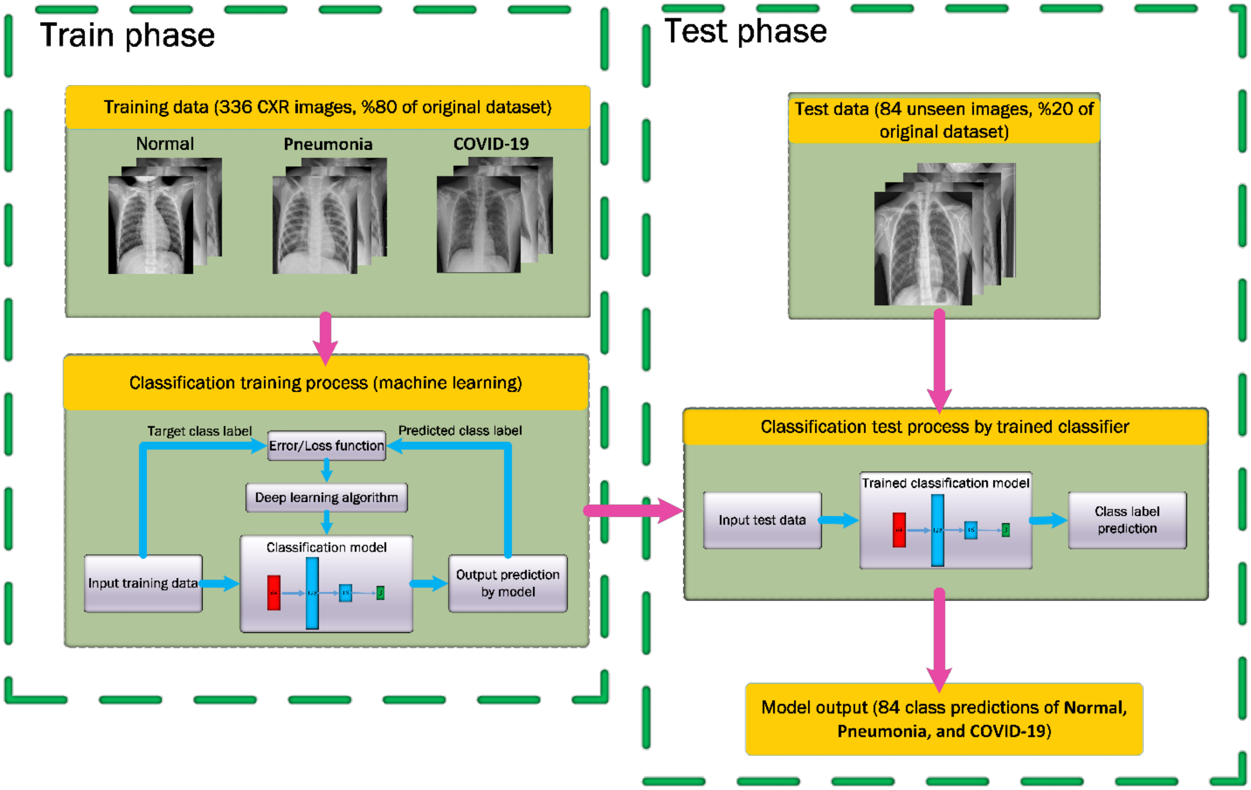

Supplementary Figure 4: Schematic diagram of the model training and test process

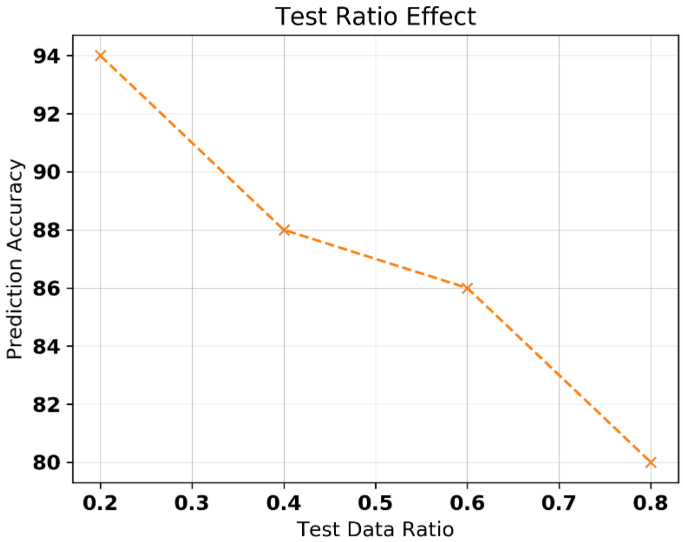

Supplementary Figure 5: An analysis of how the test ratio affects model prediction accuracy in the test phase
